# Finite Element Method Analysis of Compression Fractures on Whole-Spine Models Including the Rib Cage

**DOI:** 10.1155/2019/8348631

**Published:** 2019-05-05

**Authors:** Norihiro Nishida, Junji Ohgi, Fei Jiang, Saki Ito, Yasuaki Imajo, Hidenori Suzuki, Masahiro Funaba, Daisuke Nakashima, Takashi Sakai, Xian Chen

**Affiliations:** ^1^Department of Orthopedic Surgery, Yamaguchi University Graduate School of Medicine, 1-1-1 Minami-Kogushi, Ube, Yamaguchi 755-8505, Japan; ^2^Faculty of Engineering, Yamaguchi University, 2-16-1 Tokiwadai, Ube, Yamaguchi 755-8611, Japan

## Abstract

Spinal compression fractures commonly occur at the thoracolumbar junction. We have previously constructed a 3-dimensional whole-spine model from medical images by using the finite element method (FEM) and then used this model to develop a compression fracture model. However, these models lacked the rib cage. No previous study has used whole-spine models including the rib cage constructed from medical images to analyze compression fractures. Therefore, in this study, we added the rib cage to whole-spine models. We constructed the models, including a normal spine model without the rib cage, a whole-spine model with the rib cage, and whole-spine models with compression fractures, using FEM analysis. Then, we simulated a person falling on the buttocks to perform stress analysis on the models and to examine to what extent the rib cage affects the analysis of compression fractures. The results showed that the intensity of strain and the vertebral body with minimum principle strain differed between the spine model including the rib cage and that excluding the rib cage. The strain on the spine model excluding the rib cage had approximately twice the intensity of the strain on the spine model including the rib cage. Therefore, the rib cage contributed to the stability of the thoracic spine, thus preventing deformation of the upper thoracic spine. However, the presence of the rib cage increased the strain around the site of compression fracture, thus increasing the possibilities of a refracture and fractures of adjacent vertebral bodies. Our study suggests that the analysis using spine models including the rib cage should be considered in future investigations of disorders of the spine and internal fracture fixation. The development of improved models may contribute to the improvement of prognosis and treatment of individual patients with disorders of the spine.

## 1. Introduction

The thoracic spine consists of 12 vertebrae and attaches to the rib cage. The rib cage consists of 12 ribs each on the right and left sides. The T1 to T10 vertebrae are connected to the sternum through the ribs and costal cartilages, forming a rigid structure. For this reason, the thoracic spine is less mobile than the cervical and lumbar spines. However, the T11 and T12 vertebrae are not supported by the rib cage and are greatly mobile as discussed by White and Panjabi [1]. Because they are also the transition sites from the kyphotic curve of the thoracic spine to the lordotic curve, considerable mechanical stress is exerted on the segments from T11 to L2 vertebrae, which are considered to be common sites of compression fractures as discussed by Gertzbein [2].

In our previous study, a 3-dimensional (3D) whole-spine model was constructed from medical images by using the finite element method (FEM) and then used to develop a compression fracture model as discussed by Nakashima [3]. Then, stress analysis was performed to test whether the models were clinically relevant. However, because these models lacked the rib cage, the high degree of freedom in the thoracic spine was a problem. Thus, we did not examine the extent to which the presence of the rib cage affected the analysis of compression fractures with the whole-spine models. None of the other studies on the spine have reported analysis of compression fractures by using whole-spine models including the rib cage that were constructed from medical images. In the present study, we added the rib cage to the whole-spine models. Then, falling was simulated and the models were analyzed in the same manner as in our previous study to examine to what extent the rib cage affects the analysis of compression fractures. The analysis was performed on the assumption that the thoracic spine was stabilized by attaching the thorax and the load increased at the thoracolumbar junction.

## 2. Materials and Methods

### 2.1. Patient Images

Computed tomography (CT) images (0.6 mm slice thickness) of the whole spine, from the cervical spine to the pelvis, of an adult man (Japanese; age 32 years) were obtained with the Brilliance 64 CT scanner (Philips Healthcare, Amsterdam, Netherlands). The use of these CT images was approved by the ethics committee at the Center for Clinical Research, Yamaguchi University Hospital (Ube, Japan; approval no. H29-052).

However, this adult man had no reason to undergo chest CT. Thus, because CT involves radiation exposure, no data on the rib cage were obtained. Thus, a man in his 50s with a similar body constitution and who had been required to undergo chest CT in another clinical study was selected. A rib cage (consisting of the ribs, costal cartilages, and sternum) constructed from his CT images was added to the spine models described above. The use of these CT images was approved by the ethics committee at the Center for Clinical Research, Yamaguchi University Hospital (Ube, Japan; approval no. H28-054).

### 2.2. Model Construction

Model construction was performed with FEM analysis software (Simpleware ScanIP version M-2017.06; Synopsys Inc., Mountain View, CA, USA). After the spine was extracted, the vertebrae were mapped into the cancellous and cortical bones, and the sizes of the intervertebral discs were adjusted to match the sizes of the end plates of each vertebra.

**Figure 1:**
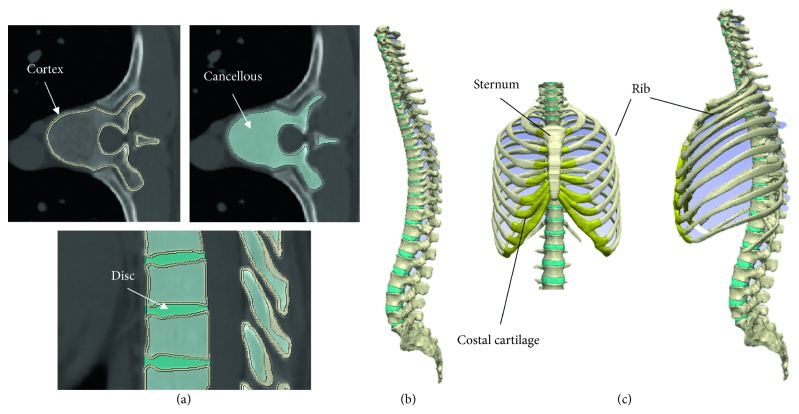
Model construction. (a) Cancellous and cortical bones and the intervertebral discs. (b) Normal spine model excluding the rib cage. (c) Normal spine model including the rib cage.

When separating the cortical and cancellous bone, the cortical bone was delineated using CT values (Hounsfield unit more than 1000) of the cortical bone. Color coding of the cancellous bone was performed on the assumption that the cancellous bone was all contained within the cortical bone. Because of the small size of the facet joints, the computer could not automatically separate them. Manual distinction was therefore made while checking the CT (Figure 1(a)). A 3D whole-spine model was constructed by individually mapping all vertebrae and intervertebral discs from the cervical to the sacral regions. The gap between each vertebra and intervertebral disc was considered to be completely restricted in movement. Facet joint spaces were created at all levels so that each vertebra could move independently (Figure 1(b)). This model was used as the normal spine model excluding the rib cage. Then, the positions of the rib cage and the spine were adjusted according to the anatomy. The thoracic vertebrae are articulated with the ribs through the costovertebral joints formed by the right and left transverse costal facets and the superior and inferior costal facets. In the model, each rib was connected to the thoracic vertebrae at one point of each transverse costal facet on the transverse process and another point formed by the superior and inferior costal facets on the vertebral bodies. Therefore, four points (the right and left side transverse process and rib and the costal facet and rib) were joined together. These regions were combined to construct a whole-spine model including the rib cage. This model was used as the normal spine model including the rib cage (Figure 1(c)).

**Figure 2:**
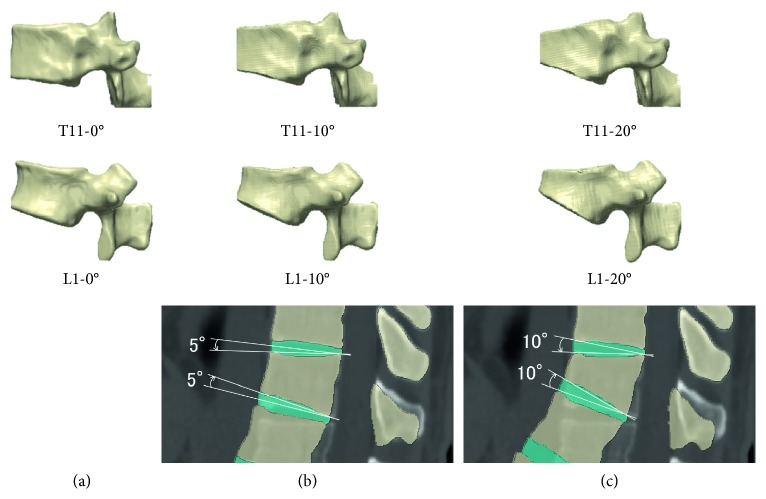
Compression fracture model construction. (a) Normal vertebral body. (b, c) T11 and L1 vertebrae trimmed by 5° and 10° to make the angle formed by the cranial and caudal surfaces of each vertebra 10° and 20°, respectively, the normal spine model excluding the rib cage.

Whole-spine models with compression fractures were created by trimming the cranial and caudal surfaces of the T11 and L1 vertebrae by 5° and 10° to make the angle formed by the cranial and caudal surfaces of each vertebra 10° and 20°, respectively, and also by rotating the intervertebral discs on the cranial and caudal sides of each vertebra (Figure 2).

**Figure 3:**
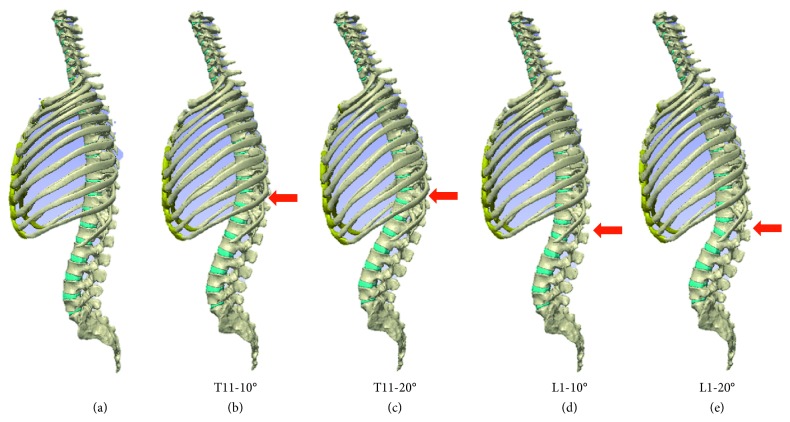
Compression fracture model construction. (a) Normal spine model including the rib cage. (b) T11-10° compression fracture model. (c) T11-20° compression fracture model. (d) L1-10° compression fracture model. (e) L1-20° compression fracture model.

These were defined as the T11-10°, T11-20°, L1-10°, and L1-20° compression fracture models (Figure 3). Considering that in normal sagittal alignment, a perpendicular line from the center of the C7 vertebra passes through the center of the upper surface of the sacral vertebrae, and the standing position was reproduced by rotating the sacral vertebrae to compensate for kyphosis.

In the normal spine model including the rib cage, the total numbers of elements and nodes were 2,159,314 and 10,890,295, respectively. In this analysis, all elements were considered to be linear elastic materials. Young's modulus was set as follows: cortical bone (spine, rib, and sternum), 12,000 MPa; cancellous bone (spine, rib, and sternum), 1500 MPa; intervertebral disc, 10 MPa; and costal cartilage, 24.5 MPa. Poisson's ratio was set as follows: cortical bone, 0.3; cancellous bone, 0.3; intervertebral disc, 0.4; and costal cartilage, 0.3, according to a previously published paper as discussed by Xia et al. [4] (Table 1). Dynamic analysis was performed by simulating a person falling on the buttocks, with load applied to the spine.

### 2.3. Load Application

Assuming that the pelvis was in a consistent position during the fall and the sacroiliac joint was fixed, a 1200 N load, corresponding to two-thirds of the body weight (60 kg) excluding the feet, was applied in a vertical direction, distributed according to the number of nodes of the whole spine as discussed by Nakashima et al. [3]. The load increase time was set at 0.0025–0.01 s. The analysis was performed using Jvision version 3.3.0 (JSOL Corporation, Tokyo, Japan) and LS-DYNA version R9.1.0 (JSOL Corporation) software.

## 3. Results

**Figure 4:**
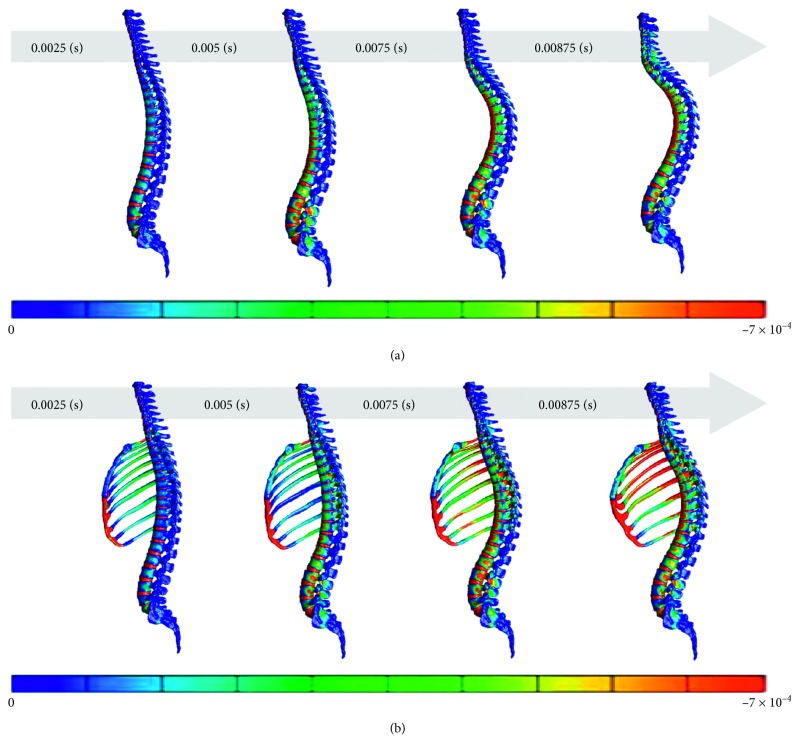
Distribution of the minimum principal strain at 0.025, 0.005, 0.0075, and 0.0875 s in the spine model. (a) Spine models excluding the rib cage. (b) Spine models including the rib cage.

Compression fracture, which is caused by compression load, was assessed in terms of the minimum principal strain. The rib cage, which is located in front of the spine, is not depicted to clearly exhibit the distribution of strain on the spine. Only the spine model excluding the rib cage was substantially deformed, presumably because its spine was not supported by a rib cage. Because of an error of abnormal element deformation, the analysis was terminated early (duration: 0.00875 s). The movement of the spine and the distribution of high-strain areas differed between the spine models including and excluding the rib cage. In the spine model including the rib cage, strain on the middle thoracic spine was more suppressed (Figure 4).

**Figure 5:**
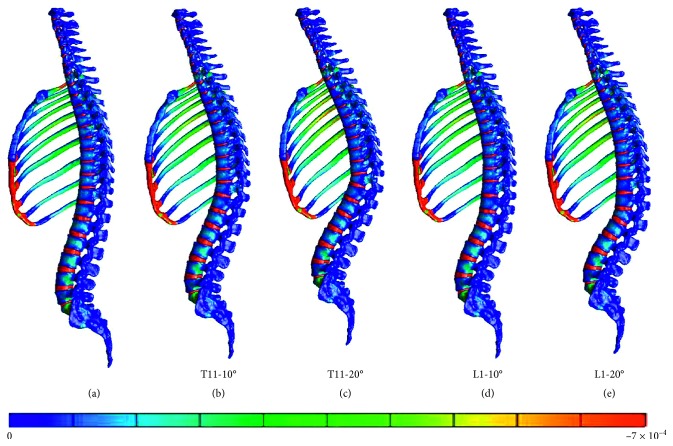
Distribution of the minimum principal strain at 0.025 s. (a) Spine models including the rib cage. (b) Compression fracture models (T11-10°). (c) Compression fracture models (T11-20°). (d) Compression fracture models (L1-10°). (e) Compression fracture models (L1-20°).

**Figure 6:**
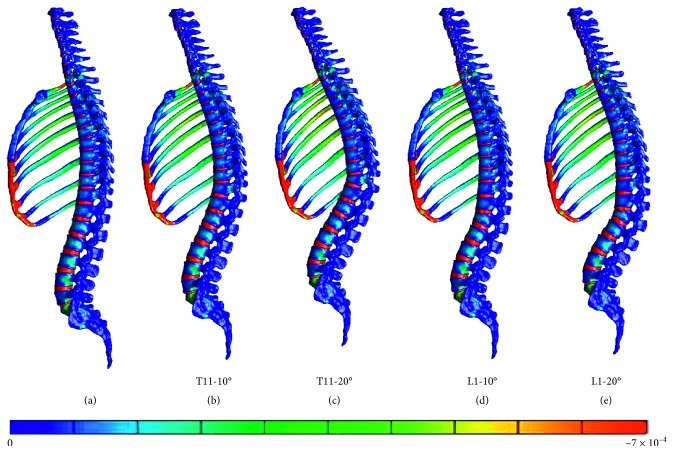
Distribution of the minimum principal strain at 0.005 s. (a) Spine models including the rib cage. (b) Compression fracture models (T11-10°). (c) Compression fracture models (T11-20°). (d) Compression fracture models (L1-10°). (e) Compression fracture models (L1-20°).

**Figure 7:**
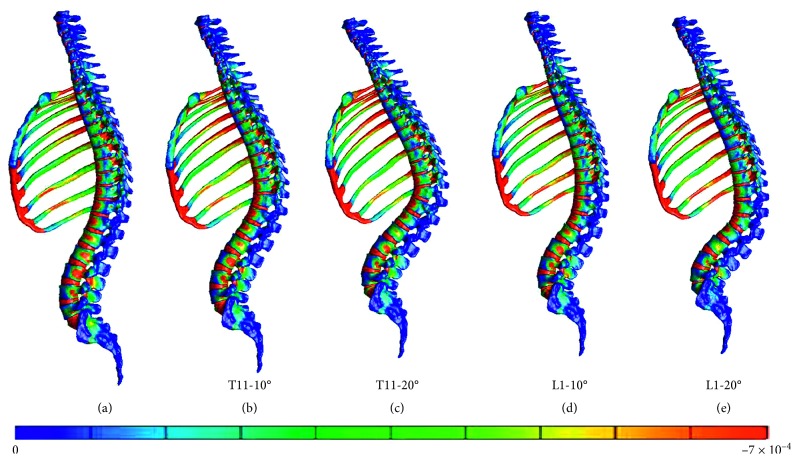
Distribution of the minimum principal strain at 0.0075 s. (a) Spine models including the rib cage. (b) Compression fracture models (T11-10°). (c) Compression fracture models (T11-20°). (d) Compression fracture models (L1-10°). (e) Compression fracture models (L1-20°).

**Figure 8:**
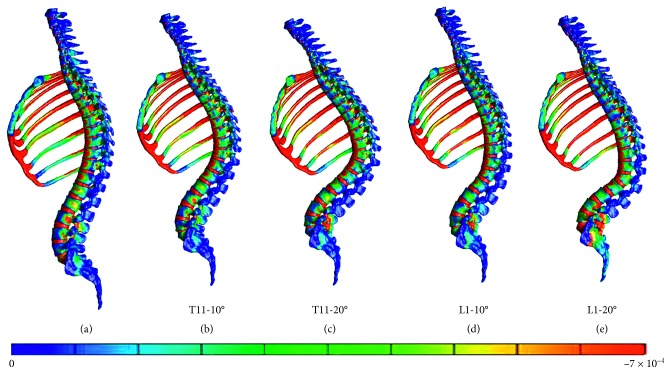
Distribution of the minimum principal strain at 0.01 s. (a) Spine models including the rib cage. (b) Compression fracture models (T11-10°). (c) Compression fracture models (T11-20°). (d) Compression fracture models (L1-10°). (e) Compression fracture models (L1-20°).

Furthermore, the vertebral fracture models including the rib cage were compared with the normal spine model including the rib cage. The figures present the distribution of the minimum principal strain at 0.025, 0.005, 0.0075, and 0.01 s in the compression fracture models (T11-10°, T11-20°, L1-10°, and L1-20°) and in the normal spine model, arranged in the order from left to right (Figures 5–8).

At 0.0025 and 0.005 s, the strain at the thoracolumbar junction increased in all models. At 0.075 and 0.01 s, strain also increased on the middle thoracic spine but occurred in different vertebral bodies. Furthermore, strain increased on the more cranial side of the fractured T11 vertebral body and on the more caudal side of the fractured L1 vertebral body.

In addition, the minimum principal strain on the middle of the ventral side of each vertebral body from T3 to L3 was plotted on graphs (at 0.0025, 0.005, 0.0075, and 0.001 s).

The intensity of strain and the vertebral body with the minimum principle strain differed between the spine models including and excluding the rib cage. The intensity was maximum on the spine model excluding the rib cage, in which some vertebral bodies (particularly the thoracic vertebrae) were affected by strain of approximately two times the intensity of those on the spine model including the rib cage. Up to the middle of the process, the minimum principle strain tended to be observed in similar vertebral bodies. However, as the process neared the end, the vertebra with the minimum principle strain moved toward the cranial direction by approximately 3 vertebral bodies in the spine model excluding the rib cage compared with the spine model including the rib cage.

In the normal spine model including the rib cage, a high-strain area first appeared at the thoracolumbar junction and then extended cranially to the lower and middle thoracic spine.

In the T11-10° and T11-20° compression fracture models, a high-strain area appeared on the fractured vertebral body and the cranially and caudally adjacent vertebral bodies at 0.025 and 0.005 s. As time progressed, the area extended cranially and caudally to the second or third vertebral bodies from the fractured vertebral body.

In the L1-10° and L1-20° compression fracture models, a high-strain area appeared on the fractured vertebral body and the cranially and caudally adjacent vertebral bodies at 0.025 and 0.005 s. As time progressed, the area extended cranially to the third vertebral body from the fractured vertebral body.

**Figure 9:**
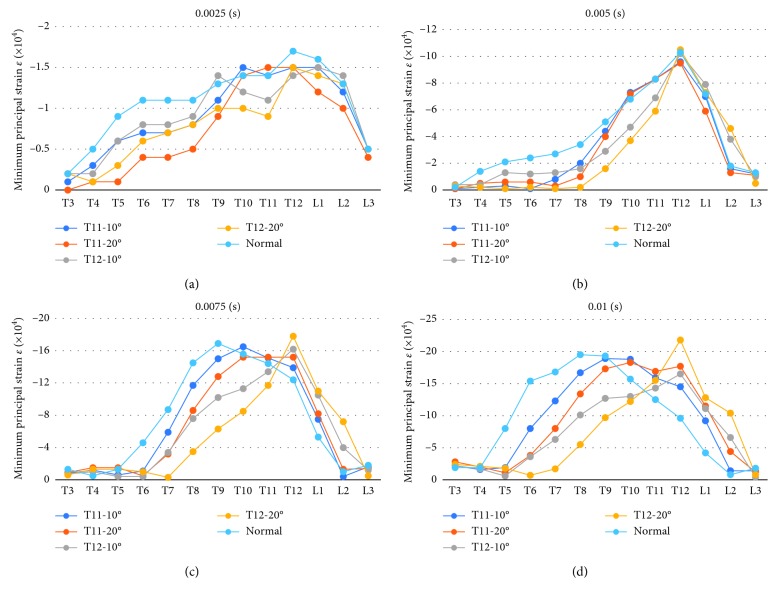
The minimum principal strain on the middle of the ventral side of each vertebral body from T3 to L3 was plotted on graphs (at the normal spine model including the rib cage, T11-10°, T11-20°, L1-10°, and L1-20° compression fracture model). (a) 0.0025 s. (b) 0.005 s. (c) 0.075 s. (d) 0.01 s.

Compared with the normal spine model including the rib cage, the spine with a preexisting vertebral fracture showed lower strain in the cranial region from the middle thoracic spine and higher strain on the fractured vertebral body (Figure 9).

## 4. Discussion

Although there have been reports of finite element analysis of the spine as discussed elsewhere [5, 6], because the facet joints are small, constructing a model of the spine from medical images requires more time than constructing models of the knee and hip joints. However, we have analyzed disorders of the spine, examined the physical properties of the organs that are necessary for simulation, and elucidated the pathology of the disorders through simulation as discussed elsewhere [7, 8]. In recent years, advances in computer technology have led to an increase in reports of analysis of the spine as discussed elsewhere [9–14]. However, there has been no published study in which whole-spine models were used. For further development of human models, it is necessary to examine how to construct models from medical images and which element should be excluded or included in constructing models mimicking the actual clinical conditions. Our objective was to perform simulation to predict prognosis in the treatment of individual patients with disorders of the spine. To achieve this objective, we investigated the effects of the rib cage by adding it to the spine models in the present study.

Spinal compression fractures commonly occur at the thoracolumbar junction (T11 to L2 levels), which is vulnerable to stress owing to the biomechanics of this region and can lead to kyphotic deformity of the spine at the fracture site. This tendency is attributed to the fact that the thoracolumbar junction is the transition point from the kyphotic curve of the thoracic spine to the lordotic curve of the lumbar spine. In FEM and cadaveric analyses as discussed elsewhere [15–18], the thoracolumbar junction has also been reported to be an origin of fractures. A study on changes in stress on the thoracolumbar junction due to postural changes has also demonstrated that stress on the region from the junction to the lumbar spine increases as the center of mass is displaced forward. On the basis of the results of the present study, a high-strain area first appeared at the thoracolumbar junction in both the spine models (including and excluding the rib cage). Because strain occurred at the thoracolumbar junction in both models, the models were consistent with those described in previous reports.

Furthermore, when a vertebral body is fractured, the risk of fracture is considered to increase by 3–5 times in the adjacent vertebral bodies as discussed by Lindsay et al. [19]. In the present study, compared with the normal spine model, the spine with a preexisting vertebral fracture also showed lower strain in the cranial region from the middle thoracic spine and higher strain mainly at the fractured vertebral body and the adjacent intervertebral discs. This suggested that when a similar load is repeatedly applied for a short period, a refracture of the previously fractured vertebral body or damage of the adjacent intervertebral discs may occur.

The reasons for the susceptibility of the thoracolumbar junction to stress include the presence of the rib cage. The researcher studied the spinal column and the rib cage with 3D mathematical models and reported that the model including the rib cage was more rigid than the model excluding the rib cage and had reduced lateroversion and rotation as discussed by Andriacchi et al. [20]. White examined the range of motion at each thoracic vertebral level and reported that during anteflexion, retroflexion, and lateroversion, the range of motion is smaller at the upper and middle thoracic spine than that at the lower thoracic spine [1]. In case of unilateral facet joint damage, the region supported by the rib cage is relatively stable, whereas the thoracolumbar junction is considered to be prone to instability as discussed elsewhere [21, 22]. Thus, the rib cage contributes to the stability of the thoracic spine. However, no study has presented any FEM analysis of the extent of the influence of the presence or absence of the rib cage on whole-spine and compression fracture models. The results of the present study showed that the intensity of strain and the vertebral body with minimum principle strain differed between the spine model including the rib cage and that excluding the rib cage, and the strain on the spine model excluding the rib cage was approximately twice as intense as the strain on the spine model including the rib cage. These findings seemed to be attributable to the fact that support from the rib cage prevented the deformation of the upper thoracic spine. The rib cage was found to contribute to the stability of the thoracic spine. Because of the presence of the rib cage, a brief stress was found to increase the strain around the compression fracture site and to amplify the possibilities of a refracture and fractures of adjacent vertebral bodies. These findings were also consistent with those described in previous clinical reports.

The present study had limitations. Because the degree of freedom of the ribs remains undetermined, the stress on them was increased. This increased stress might not cause a rib fracture. Thus, further studies are needed to determine whether the increased stress was due to the flexibility of the ribs and to assess the conditions of the costovertebral joints. The present models did not consider ligaments, joint capsules, and muscles. The change of alignment associated with spinal compression fractures was compensated for only by rotation of the pelvis. Also, the analysis did not consider the patient's posture, the hardness of the ground, or the elapsed time during the fall. Finally, the material constants of the vertebrae and discs and ribs are fixed.

Nevertheless, the findings obtained from FEM models of the whole-spine model including the rib cage in this study support previous findings. The development of models from medical images may contribute to the prevention of damage to the vertebrae and intervertebral discs and to the development of the therapy of conservation using corset, rehabilitation programs, and operation plans.

## 5. Conclusions

In this study, FEM models of the whole spine including the rib cage were created from medical images, and strain analysis was performed using compression fracture models. The results showed that addition of the rib cage increased the stability of the thoracic spine and that the thoracolumbar junction was more susceptible to fractures in the whole-spine model including the rib cage and in the compression fracture models. When disorders of the spine and internal fracture fixation are simulated in the future, analysis using spine models including the rib cage should be considered.

## Figures and Tables

**Table 1:** Young's modulus, Poisson's ratio, and mass density.

Part		Young's modulus *E* (MPa)	Poisson's ratio, *ν*	Mass density, *ρ* (g/cm^3^)
Spine	Cortical bone	12000	0.3	1.56
	Cancellous bone	1500	0.3	0.29

Intervertebral disc		10	0.4	1.0
Rib	Cortical bone	12000	0.3	1.56
	Cancellous bone	1500	0.3	0.29

Costal cartilage		24.5	0.3	1.5
Sternum	Cortical bone	12000	0.3	1.56
	Cancellous bone	1500	0.3	0.29

## Data Availability

The medical image data used to support the findings of this study are restricted by the ethics committee at the Center for Clinical Research, Yamaguchi University Hospital, in order to protect patient privacy.
